# Unexpected discovery of a new *Podonychus* species in Kyushu, Japan (Coleoptera, Elmidae, Elminae, Macronychini)

**DOI:** 10.3897/zookeys.933.48771

**Published:** 2020-05-18

**Authors:** Hiroyuki Yoshitomi, Masakazu Hayashi

**Affiliations:** 1 Entomological Laboratory, Faculty of Agriculture, Ehime University, Tarumi 3-5-7, Matsuyama, 790-8566, Japan Ehime University Tarumi Japan; 2 Hoshizaki Green Foundation, Sono, Izumo, 691-0076, Japan Hoshizaki Green Foundation Sono Japan

**Keywords:** disjunct distribution, endophallus, larvae, new species, riffle beetle, SEM, taxonomy

## Abstract

*Podonychus gyobu***sp. nov.**, a second species of the genus *Podonychus* Jäch & Kodada, 1997, hitherto known only from Indonesia, is described from Kyushu, Japan. This new species is similar to *P. sagittarius* Jäch & Kodada, 1997, but differs from it in the straight penis, arcuate 2^nd^ labial palpomere, and in the 3^rd^ antennomere being longer than wide. The endophallic structures and the larva of *P. gyobu***sp. nov.** are described. A character matrix of the Macronychini genera and a key to the Japanese genera are provided.

## Introduction

The riffle beetle fauna of Japan is well studied and 17 genera with 57 species are reported so far (Kamite et al. 2018). Some undescribed species and taxonomic problems still remain (Kamite et al. 2018; Nakajima et al. 2020). The larval stages of the Japanese species are well known and except for a few taxa, most larvae were described recently (Yoshitomi and Satô 2005; Hayashi 2009, 2013; Hayashi and Sota 2010, 2015, 2019; Hayashi and Yoshitomi 2014, 2015; Kamite 2015; Hayashi et al. 2016, 2019).

The genus *Podonychus* Jäch & Kodada, 1997 (Elminae, Macronychini) was hitherto known only from Siberut Island, Indonesia (Jäch et al. 2016). It has been regarded as monotypic so far. This genus is peculiar in having 6-segmented antennae, which is the smallest number of antennomeres within the family Elmidae (Jäch and Kodada 1997; Kodada and Jäch 2005). Unexpectedly in 2018, some specimens of this genus were collected in Kyushu, Japan. After repeated field investigation and closer examination, it was clear that the specimens represent a new species, closely related to *P. sagittarius* Jäch & Kodada, 1997.

In the present paper, we describe this new species including endophallic structures and the larva.

## Material and methods

Adults and larvae were collected from rivers by using small hand nets. The larval determination was done by association and by rearing. In Iroha-gawa (river) eight elmid species were collected (*Podonychus gyobu* sp. nov.; *Leptelmis gracilis* Sharp, 1888; *Stenelmis nipponica* Nomura, 1958; *S. vulgaris* Nomura, 1958; *Grouvellinus nitidus* Nomura, 1963; *Zaitzevia awana* (Kôno, 1934); *Zaitzeviaria ovata* (Nomura, 1959); *Z. brevis* (Nomura, 1958)); the larvae of all these species, except *Podonychus gyobu*, are included in the key by Hayashi and Sota (2010); MH has reared one couple of adults, and got an immature larva of F1 generation of this species.

The holotype and some paratypes will be deposited in the Ehime University Museum, Matsuyama, Japan (**EUMJ**), and the remaining paratypes in the Naturhistorisches Museum Wien, Austria (**NMW**), the Kitakyushu Museum of Natural History and Human History, Kitakyushu, Japan (**KMNH**), and the Hoshizaki Institute for Wildlife Protection, Izumo, Japan (**HOWP**).

Morphological terms used to describe the genitalia and the larva follow Kodada and Jäch (2005) and Hayashi et al. (2019).

General observations and dissections were made under a stereoscopic microscope (Leica MZ95). Microstructures of the dissected parts were mounted on hollow slides with pure glycerine and observed under a microscope (Olympus BH-2). After observation, the dissected parts were mounted on slides with Canada Balsam. SEM photographs were taken using a scanning electron microscope (JCM-6000 Neoscope; JEOL Ltd., Tokyo, Japan).

Morphological abbreviations used in this study are as follows:

EL length of elytra

PW width of pronotum

EW width of elytra

PL length of pronotum

TL total length (EL + PL)

The average measurement is given in parentheses after the range. For male genitalia, we made the following measurements after Hayashi and Yoshitomi (2015, fig. 1):

BL basal length of penis, from base to the point where MH and ML lines meet

C degree of angle at MH point, formed by LBP and LCP lines crossing

CL caudal length of penis (= ML−BL)

LB length of phallobase

LBP length of basal portion, line connecting base with MH point

LCP length of caudal portion, line connecting MH point with apex

MH maximum height of penis, vertical line from ML line to C point

ML maximum length of penis (= BL+CL)

## Taxonomy

### Podonychus

Taxon classificationAnimaliaColeopteraElmidae

Jäch & Kodada, 1997

F54767BE-547A-5AF4-8A47-FCBFE76BCEBF

#### Type species.

*Podonychus sagittarius* Jäch & Kodada, 1997

#### Diagnosis.

The genus is very distinctive in having the following characteristics of the adults (see also Table 1): 1) antennae 6-segmented; 2) pronotal median groove very thin and shallow; 3) pronotal sublateral carinae absent; 4) lateral declivity of the pronotum with oblique impressions; 5) each elytron with one sublateral carina on interval VIII. The larva is also distinctive in having Y-shaped projections (Fig. 7A, E, D) on the thorax and abdomen.

**Table 1.** Character matrix of Macronychini genera. Abbreviations: parameres (a = absent; p = present); basal piece (s: short; l: long); hind wing (B: brachypterous; M: macropterous; MI: micropterous).

Genus	Antennal segment no.	Parameres	Labial palpo-meres no.	Basal piece	Hind wing	Elytral carinae	Distribution	References
* Eonychius *	10	a	3	s	B	V-VII-VIII	China (Hong Kong)	Jäch and Boukal (1995)
* Vietelmis *	10	p	3	s	M	V-VII, V-VII-VIII	Vietnam, China, Borneo, Laos, Thailand, Malaysia	Kodada et al. (2017)
* Haraldaria *	9	a	3	s	B	V-VI-VII	Malaysia	Jäch and Boukal (1996)
* Homalosolus *	9	a	3	l	M/B	V-VII-VIII, III-V-VII-VIII	Borneo	Jäch and Kodada (1996a)
* Aulacosolus *	8	a	2	s	M	V-VII, III-V-VII, III-V-VII-VIII	India, Thailand, Laos, Malaysia, Indonesia (Sumatra)	Jäch and Boukal (1997)
* Cuspidevia *	8	p	3	s	M	VII	China	Jäch and Boukal (1995)
* Graphosolus *	8	a	2	s	B	III-V-VII-VIII	Borneo, Indonesia (Sumatra, Java), Malaysia, Philippines	Jäch and Kodada (1996b)
* Indosolus *	8	a	2	s	M	V-VI-VII	Myanmar, India, China, Malaysia	Jäch and Boukal (1995)
* Jilanzhunychus *	8	a	3	s	B	III-V-VII-VIII	China	Jäch and Boukal (1995)
* Loxostirus *	8	a	2	s	B	III-V-VII-VIII	Borneo	Jäch and Kodada (1996a)
* Macronevia *	8	p	3	s	M/B	V	Malaysia	Jäch and Boukal (1996)
* Nesonychus *	8	a	2	s	B	V-VII	Borneo, Indonesia (Sumatra, Java, Lombok)	Jäch and Boukal (1997)
* Okalia *	8	a	3	s	M/B	V-VI-VII	Malaysia	Kodada and Čiampor (2003)
* Paramacronychus *	8	p	3	s	B	III-V-VII-IX, V-VII-IX	Japan	Jäch and Boukal (1995)
* Rhopalonychus *	8	a	3	s	M	VII-VIII	Borneo	Jäch and Kodada (1996a)
* Urumaelmis *	8	p	3	s	M	V-VI	Japan	Jäch and Boukal (1995)
* Zaitzevia *	8	p/a	3	s	M/B	V-VI-VII, V-VII-VIII	India to China, Japan, Korea, Russian Far East, North America	Jäch and Boukal (1995)
* Zaitzeviaria *	8	p/a	3	s	M/B	VII-VIII	East Palearctic and Oriental Regions	Hayashi and Yoshitomi (2015)
* Macronychus *	7	a	3	s	M/B	III-V-IX	Europe, North Africa, Russian Far East, China, Korea, Oriental Region, North America	Jäch and Boukal (1995)
* Prionosolus *	7	a	2	s	M/B	V-VII	Philippines, Borneo, Indonesia (Siberut)	Jäch and Kodada (1997)
* Sinonychus *	7	a	2	s	B	III-V-VI-VII, V-VI-VII	China, Japan	Jäch and Boukal (1995)
* Podonychus *	6	a	2	s	MI	VIII	Indonesia (Siberut), Japan	Jäch and Kodada (1997)

### Podonychus gyobu sp. nov.

Taxon classificationAnimaliaColeopteraElmidae

3A0E5C0D-67F6-556F-A246-736245938085

http://zoobank.org/3EDFF62A-25F7-4BEF-AA92-88FF2ED8B260

Figs 1 , 2 , 3 , 4 , 5 , 6 , 7 , 8 Japanese name: Hyotan-hime-doromushi

#### Type material.

***Holotype*** (EUMJ): male, “JAPAN: KYUSYU/Oita Pref. Usa shi/Yamabukuro: Iroha-gawa”, “[locality name in Japanese characters] 12. XII. 2018 33.498440, 131.28958 D. Inoue leg.”. ***Paratypes*** (EUMJ, NMW, HOWP, KMNH), 1 ex., same data as for the holotype; 1 ex., same locality, but 8. XII. 2018; 2 males, 2 females, and 6 exs., same locality, but 15. XII. 2018, J. Nakajima, D. Inoue leg.; 6 exs., same locality, but 30. III. 2019, H. Yoshitomi leg.; 1 ex., “JAPAN: KYUSYU Oita Pref. Usa-shi [locality name in Japanese characters]”, “[locality name in Japanese characters] 33.421472, 131.357887 24. XII. 2018, D. Inoue leg.”; 3 exs., “Noyama, Ajimu machi, Usami-shi, Oita Pref., Kyushu, Japan, 33.412806, 131.349694, 29. XI. 2019 H. Yoshitomi leg.”; 2 exs., “Yasaka-gawa, Yamaga-machi, Kitsuki-shi, Oita Pref., 33.430687, 131.477928, 24. I. 2020, D. Inoue leg.”.

#### Larval specimens examined.

3 mature larvae (EUMJ in ethanol), Iroha-gawa, Yamabukuro, Usa shi, Oita Pref., 30. III. 2019, H. Yoshitomi leg.

#### Comparative specimen examined.

*Podonychus sagittarius* Jäch & Kodada, 1997: 1 male paratype (EUMJ), “INDONESIEN 1991 (22) Siberut, Toteburu-Bakeuluk leg. Jäch 17.2”, “PARATYPUS Podonychus sagittarius sp. nov. des. Jäch & Kodada ‘98”.

#### Diagnosis.

The new species is morphologically similar to *P. sagittarius*, and differs from it in the following characteristics: 1) penis almost straight from base to near apex in lateral view (slightly curved ventrally in *P. sagittarius*); 2) lateral margins of the 2^nd^ labial palpomeres strongly arcuate (weakly arcuate in *P. sagittarius*); 3) 3^rd^ antennomeres longer than wide (as long as wide in *P. sagittarius*). The endophallicstructures of *P. sagittarius* were not described in detail, but judging from the aedeagus illustrations (Jäch and Kodada 1997, figs 78–80) they are similar to those of *P. gyobu* sp. nov.

#### Description of adult.

**Figure 1.**
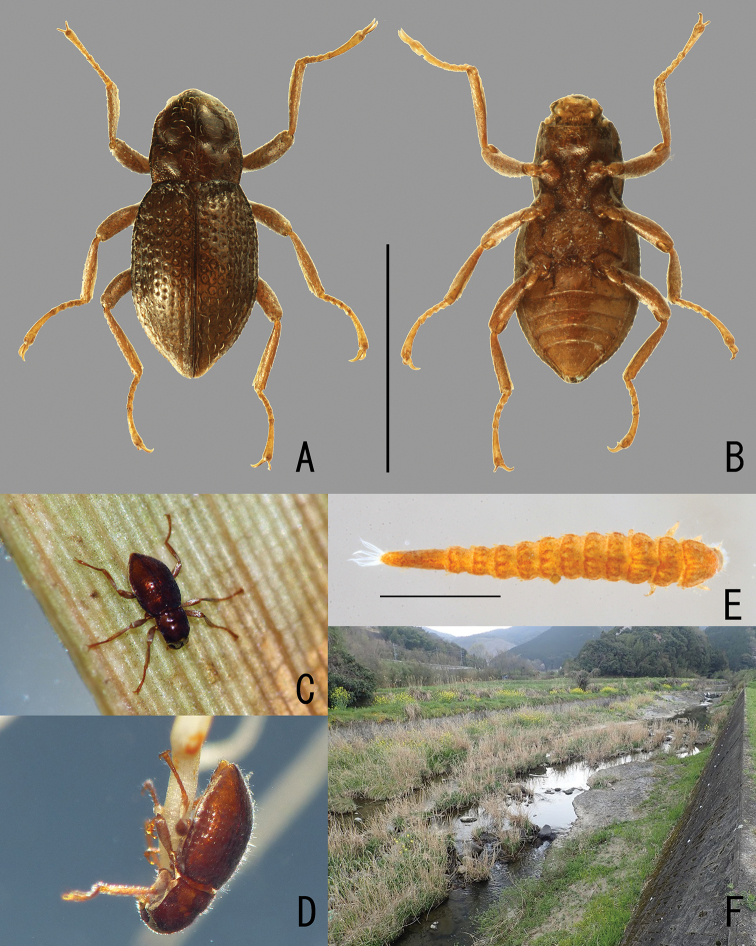
*Podonychus gyobu* sp. nov. **A, B** holotype in dorsal (**A**) and ventral view (**B**) **C, D** living adults **E** living larva **F** habitat (Iroha-gawa). Scale bars: 1.0 mm.

**Figure 2.**
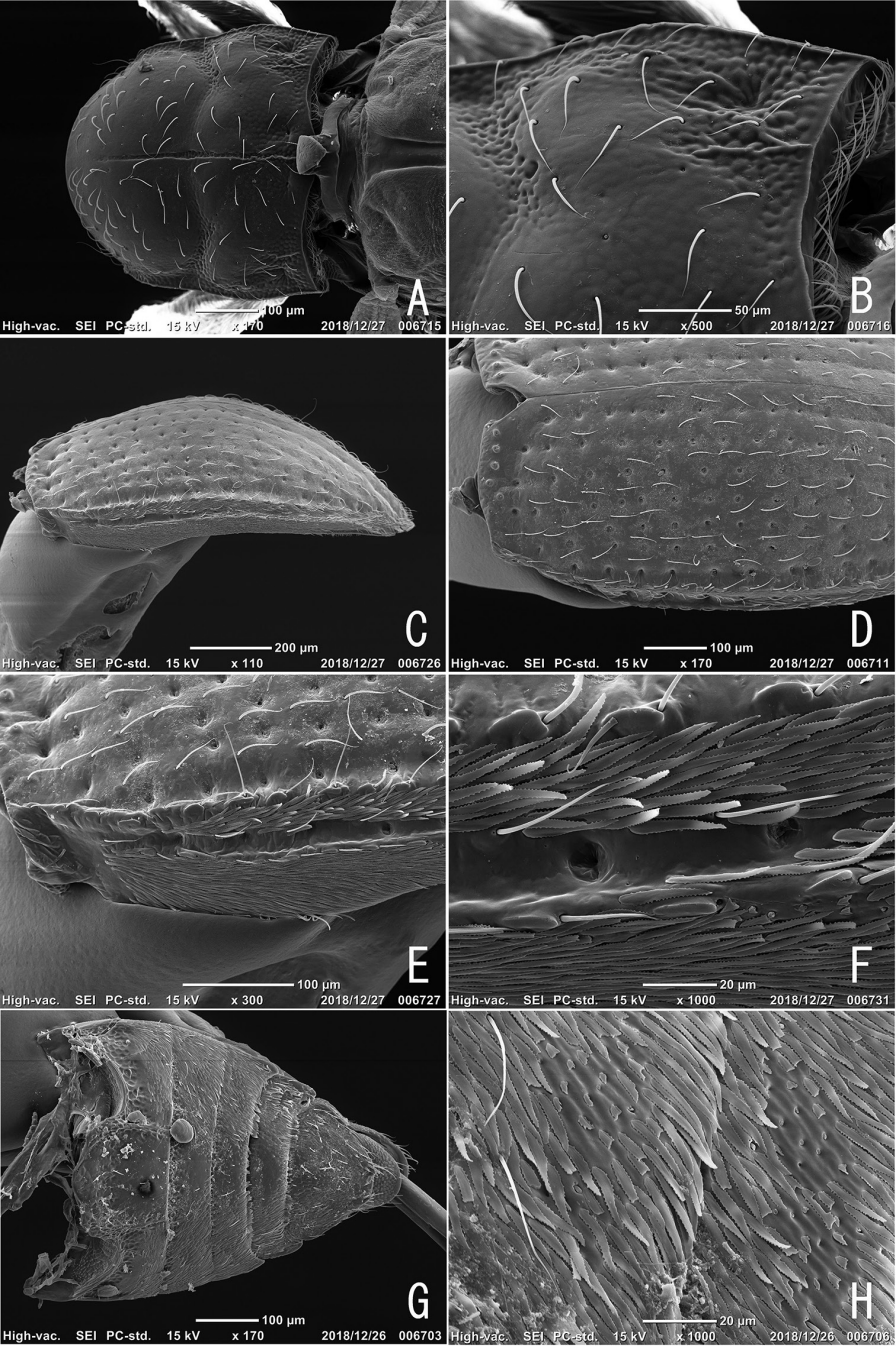
Adult of *Podonychus gyobu* sp. nov. **A** pronotum **B** close up of basal part of pronotum **C** elytra in lateral view **D** basal part of elytra in dorsal view **E** lateral part of elytra **F** close up of elytral plastron setae **G** abdominal ventrites **H** abdominal plastron setae.

**Figure 3.**
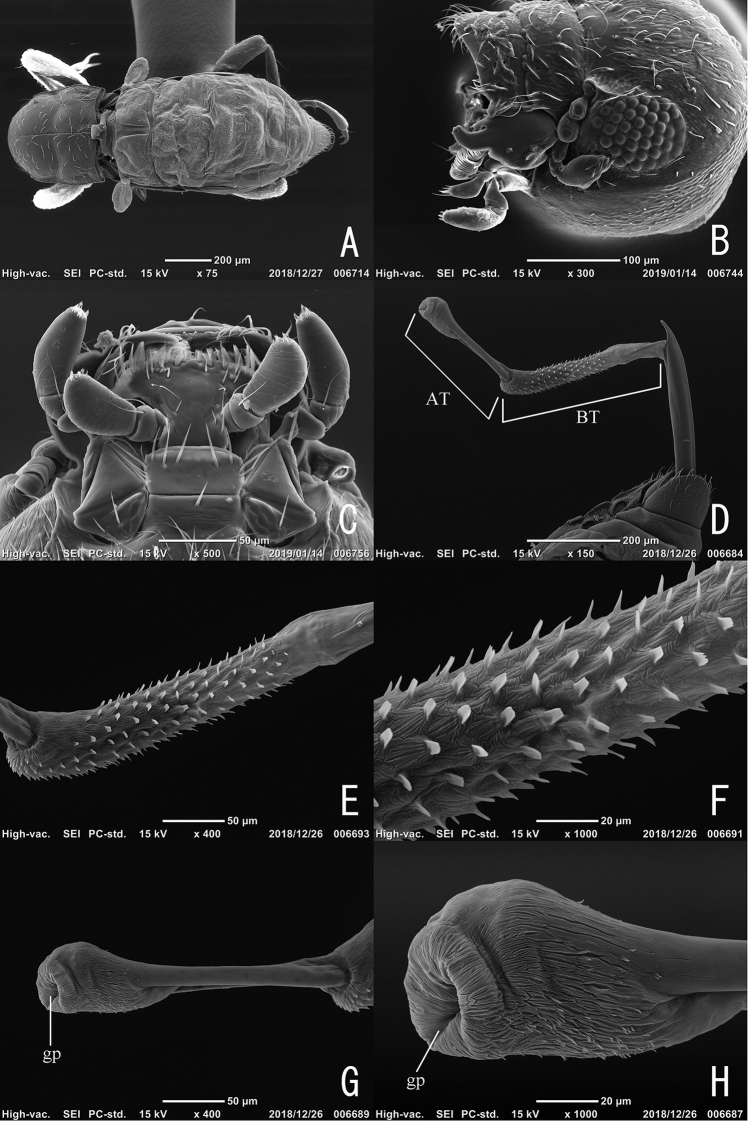
Adult of *Podonychus gyobu* sp. nov. **A** dorsal habitus without elytra **B** head in lateral view **C** labium **D** aedeagus with everted endophallus **E** basal tube of endophallus **F** spines on basal tube of endophallus **G** apical tube of endophallus **H** ditto, close up. AT: apical tube; BT: basal tube; gp: gonopore.

Body (Fig. 1A–D) obovate, convex dorsally, shiny. Coloration of body brown, mouth parts, antennae and legs paler. Plastron distributed in posterior part of head (Fig. 3B), elytral intervals VIII–IX (Fig. 2C–F), hypomera, epipleura, lateral part of meso- and metaventrites, and abdominal ventrites I–V (Fig. 2G, H).

**Figure 4.**
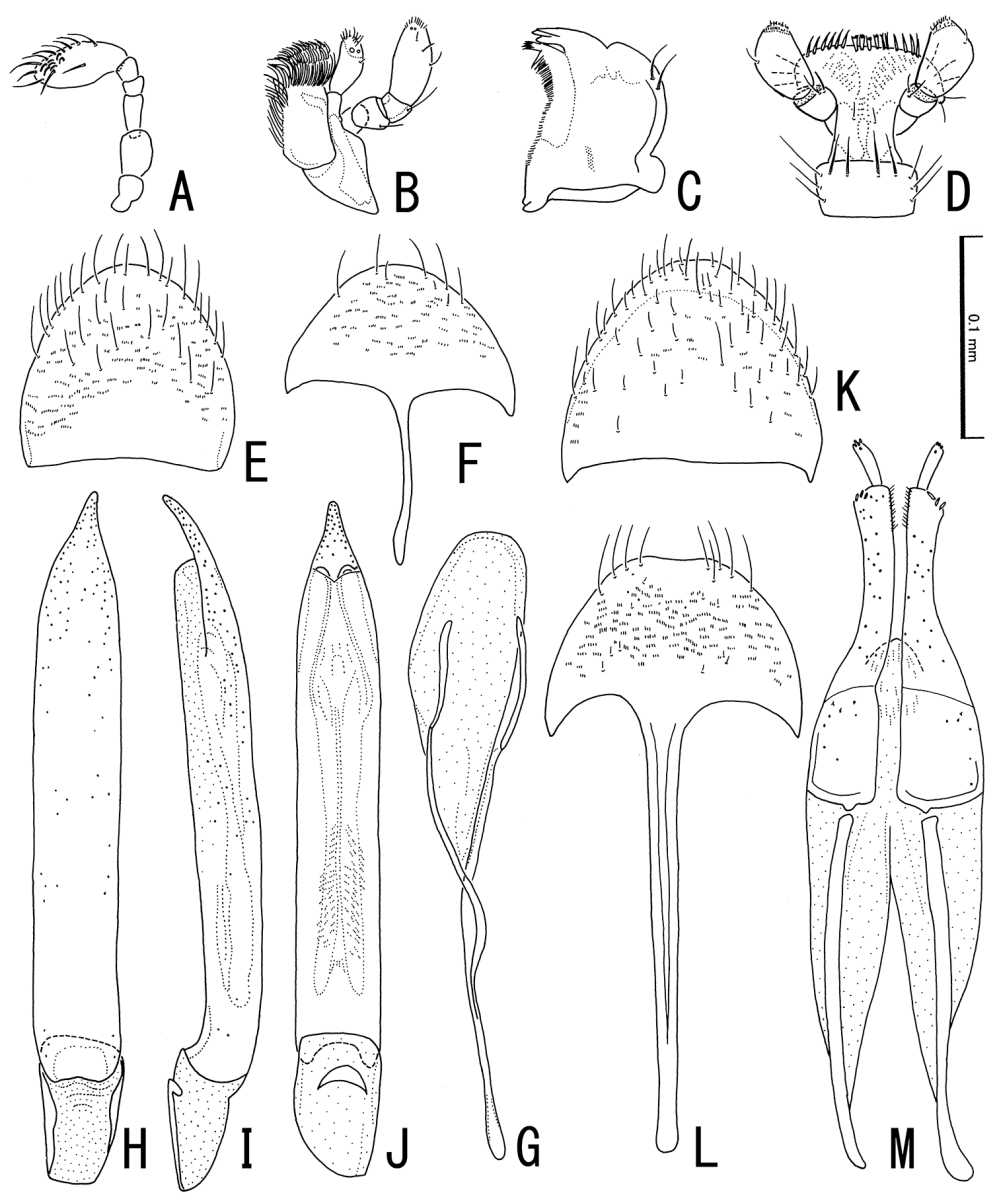
Adult of *Podonychus gyobu* sp. nov. **A–J** male **K–M** female **A** antenna **B** maxilla **C** mandible **D** labium **E, K** tergite VIII **F, L** sternite VIII **G** sternite IX **H–J** aedeagus in ventral (**H**), lateral (**I**) and dorsal views (**J**) **M** ovipositor. Scale bar: 0.1 mm.

Head with sparse suberect setae. Eyes small with about 40 facets. Antennae 6-segmented; approximate ratio of length of antennomeres 1–6: 6 : 7 : 6 : 2 : 3 : 14 (*N* = 1, paratype). Maxillae (Fig. 4B) with oblong terminal segment of galea; maxillary palpi 3-segmented, with oblong terminal palpomere. Mandibles (Fig. 4C) feebly asymmetrical, with three apical teeth, shallowly notched in antero-lateral parts, bearing setae in lateral part. Labium (Figs 3C, 4D) with 2-segmented palpi; terminal palpomeres strongly arcuate along lateral margins. Pronotum (Fig. 2B, C) with sparse suberect setae, widest at basal 1/3, depressed transversally in the middle; median groove (Fig. 2A) thin and shallow, running from posterior almost to anterior margin; sublateral carinae absent; lateral declivity (Fig. 2B) with oblique impressions; PW/PL 0.88–1.08 (1.03). Elytra with small tubercles along anterior margin, with one pair of sublateral carinae on interval VIII (Fig. 2E, F); plastron setae on intervals VIII–IX; EL/EW 1.47–1.63 (1.55); EL/PL 1.95–2.32 (2.18); EW/PW 1.28–1.50 (1.37); TL/EW 2.13–2.37 (2.26). Hind wings (Fig. 3A) reduced, examined specimen (paratype) micropterous. Legs relatively long; length of foreleg about 0.9 times as long as TL. Ventral side of thorax and abdomen more or less as in *P. sagittarius*.

**Male.** Tergite VIII (Fig. 4E) semicircular, bearing long setae in caudal part; sternite VIII (Fig. 4F) semicircular, with slender and relatively long median strut; sternite IX (Fig. 4G) slightly sclerotized, oblong, with long and slender paraproct. Aedeagus (Fig. 4H–J) long, almost straight; phallobase short, slightly less than 0.25 times as long as penis (LB/ML), with semicircular sclerotization projecting dorsally; parameres absent; penis long, almost straight, with pointed apex curved ventrally; fibula and corona absent; C 160^o^; MH/ML 0.16; BL/CL 0.60; LCP/LBP 1.59. Endophallus in everted condition (Fig. 3D–H) longer than penis, lacking bladders and distinct sclerites; basal tube (BT in Fig. 3D) long, 350 μm, bearing scaly spines; apical tube (AT in Fig. 3D) 250 μm, projecting from ventro-apical part of BT, well sclerotized and slender in basal 2/3, expanded and membranous in apical 1/3 closely covered with shallow furrows; gonopore situated at apex of AT.

**Female.** Tergite VIII (Fig. 4K) semicircular, bearing setae of variable length; sternite VIII (Fig. 4L) shallowly concave in caudal margin, bearing long setae in postero-lateral parts, with long and stout median strut. Ovipositor (Fig. 4M) longer than sternite VIII, bearing minute apical sensilla on stylus and coxite; approximate ratio of stylus, distal part of coxite, basal part of coxite and valvifer (N = 1) as 1.0 : 4.3 : 2.6 : 8.0. Secondary sexual dimorphism not strongly pronounced.

Measurement (*N* = 10). TL 1.21–1.36 (1.28) mm; PW 0.37–0.45 (0.41) mm; PL 0.37–0.43 (0.40) mm; EL 0.82–0.95 (0.87) mm; EW 0.51–0.60 (0.56) mm.

#### Description of larva.

**Figure 5.**
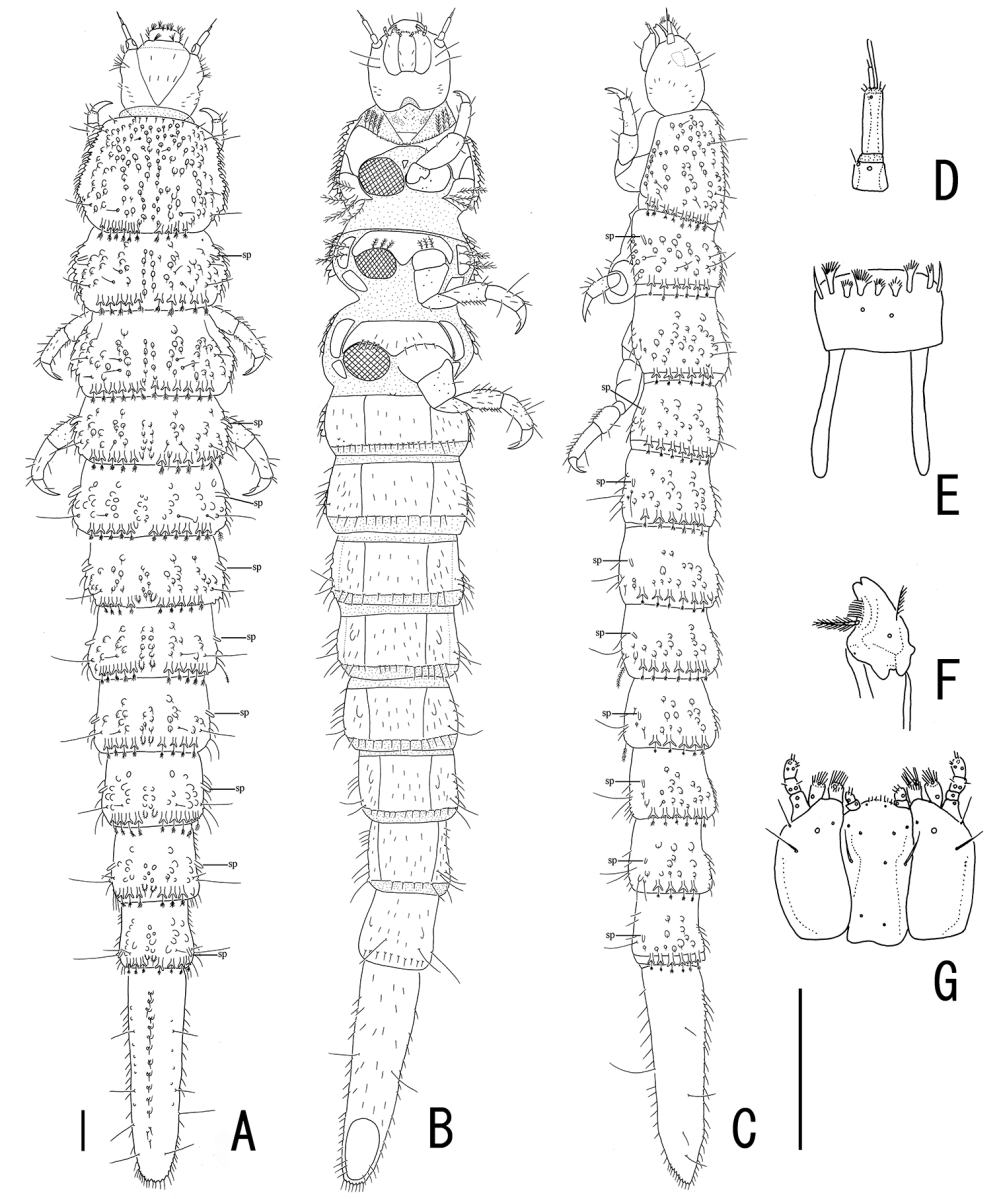
Larva of *Podonychus gyobu* sp. nov. **A–C** habitus in dorsal (**A**) ventral (**B**) and lateral views (**C**) **D** antenna **E** labrum **F** mandible **G** maxillae and labium. sp: spiracle. Scale bars: 0.1 mm.

Body (Fig. 5A–C) semi-cylindrical in cross-section, convex dorsally, flat ventrally. Coloration of body entirely pale orange; legs paler; granules on dorsum darker, forming longitudinal stripes.

**Figure 6.**
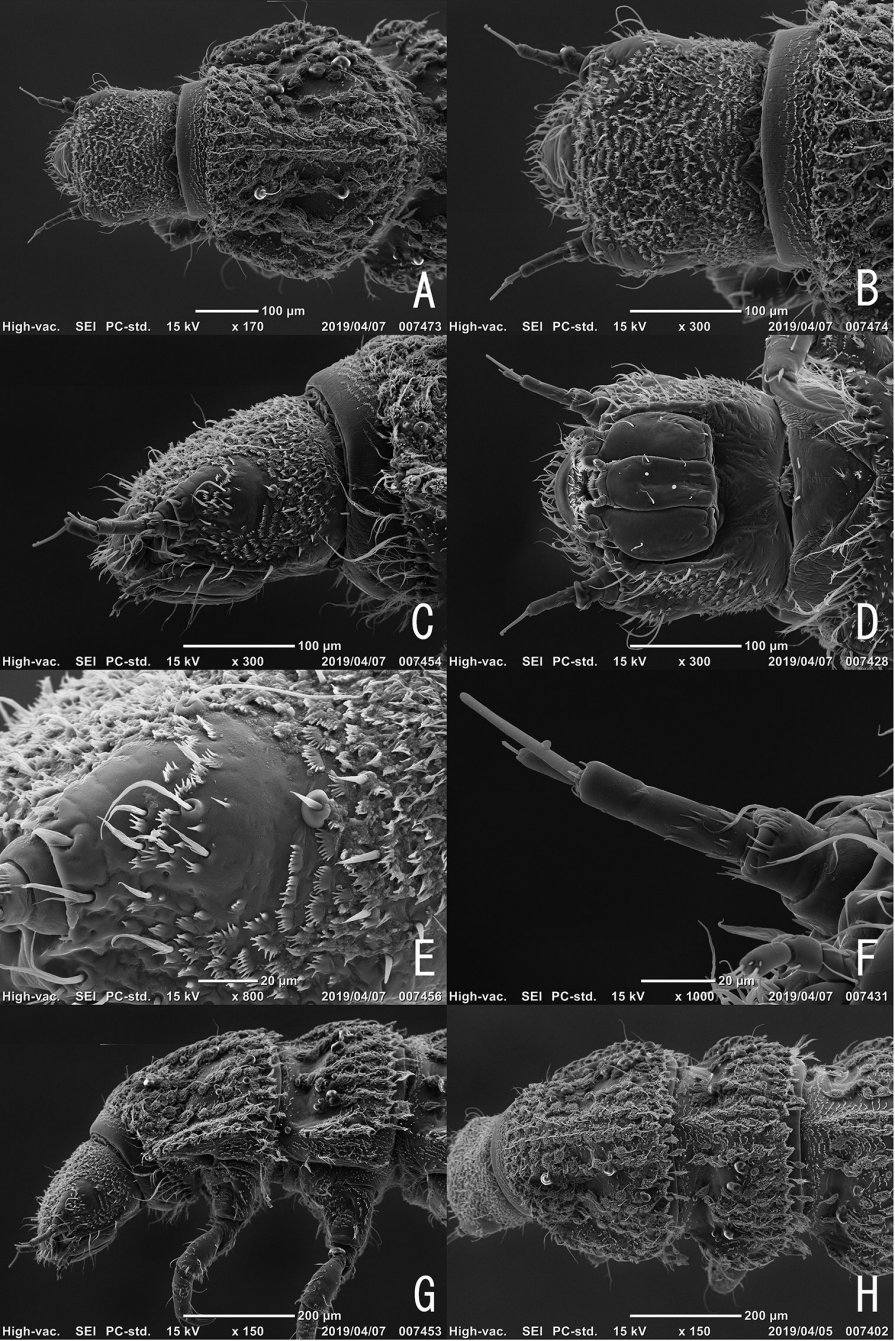
Larva of *Podonychus gyobu* sp. nov. **A** head and pronotum **B** head in dorsal view **C** head in lateral view **D** head in ventral view **E** eye area of head **F** antenna **G** pro- and mesothorax in lateral view **H** ditto in dorsal view.

**Figure 7.**
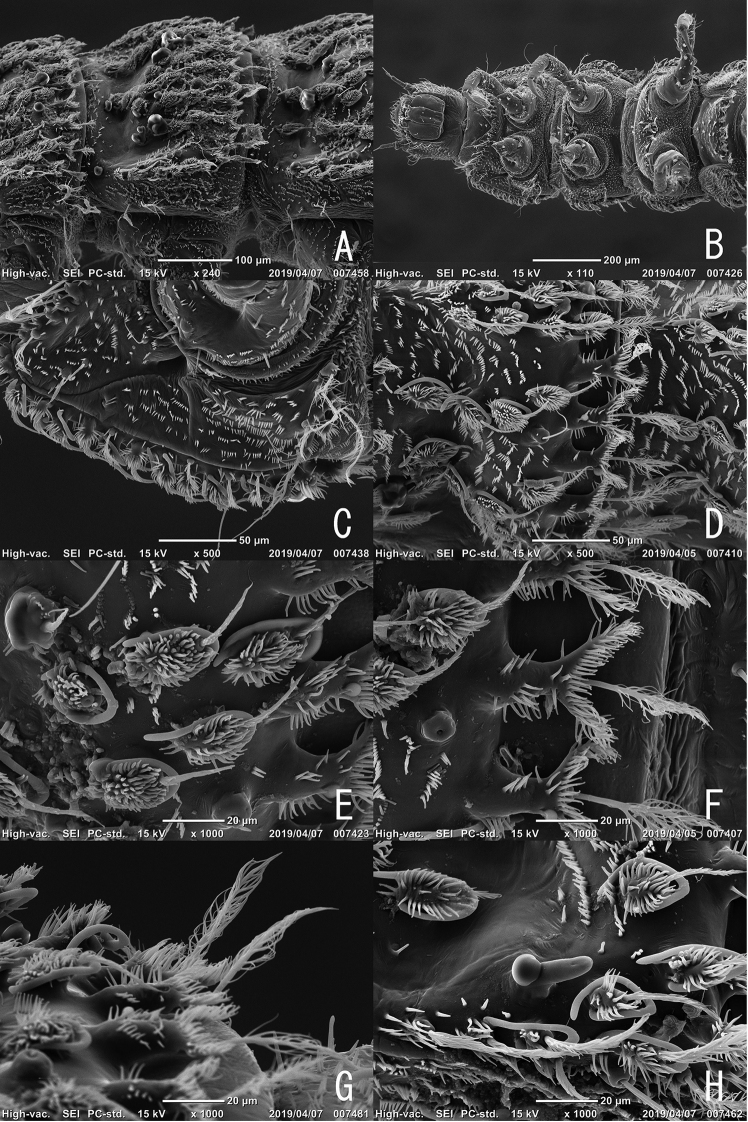
Larva of *Podonychus gyobu* sp. nov. **A** thorax in lateral view **B** ditto in ventral view **C** ventral sclerites of prothorax **D** granules on abdomen **E** ditto, close up **F** Y-shaped projection on abdomen **G** pectinate setae on postero-lateral corner of abdomen **H** spiracle on abdomen.

**Figure 8.**
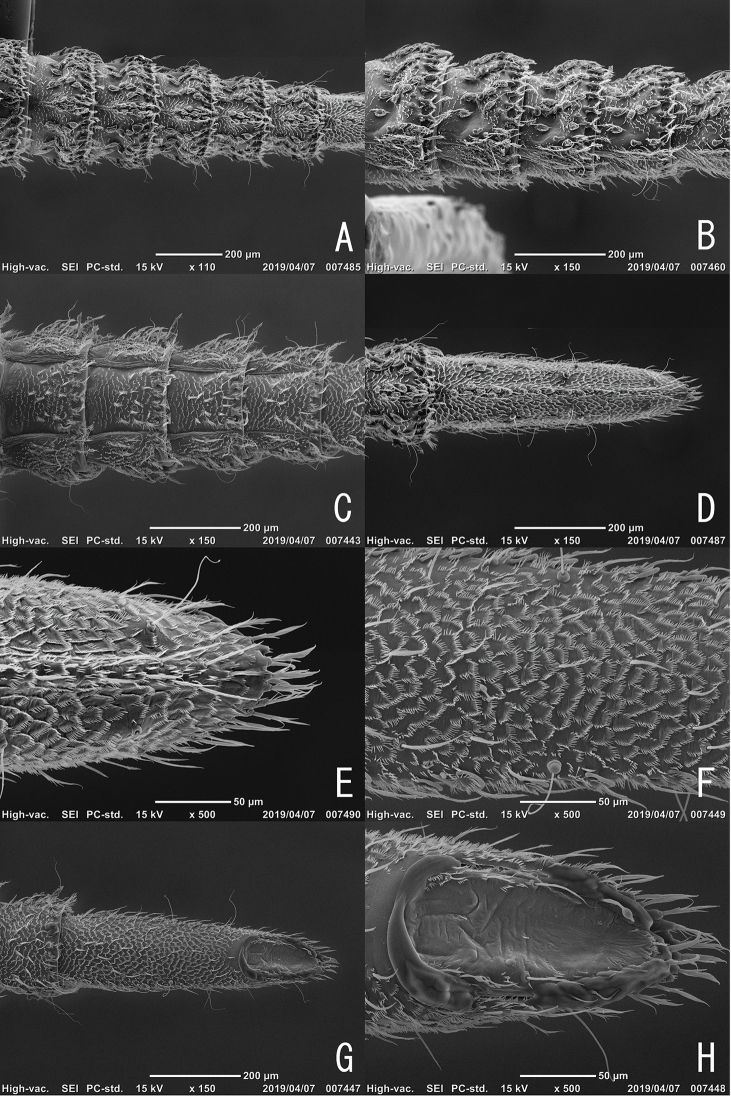
Larva of *Podonychus gyobu* sp. nov. **A** abdominal segments IV–VIII in dorsal view **B** abdominal segments IV–VIII in lateral view **C** abdominal segments V–IX in ventral view **D** abdominal segments VIII–IX in dorsal view **E** apex of abdominal segment IX in dorsal view **F** surface of abdominal segment IX in lateral view **G** abdominal segments VIII–IX in ventral view **H** operculum.

Head visible dorsally, well exposed from prothorax, trapezoidal, widest at apical 1/3, densely covered with short spines, bearing short setae. Eyes (Fig. 6C, E) lacking lens of stemmata. Antennae (Figs 5D, 6F) relatively long; antennomere Ias long as wide; antennomere II long, with long and slender sensorial appendage; antennomere III shorter than sensorial appendage on antennomere II, with short sensorial appendage at apex. Labrum (Fig. 5E) transverse, with a row of pectinate setae in anterior part of dorsal surface, with a pair of long apodemes projecting from postero-lateral corners. Mandibles (Fig. 5F) subtriangular, with long and pectinate setae on lateral parts; two apical teeth short and blunt; basal setose processes long.Maxillae (Figs 5G, 6D) with relatively long palpi. Labium (Figs 5G, 6D) with a pair of long setae in middle. Thorax serrate in lateral parts, widest at metathorax. Prothorax with seven ventral sclerites; mesal one small, situated between procoxae; antero-lateral ones wide, bearing plumose setae on anterior margin; postero-lateral ones quadrate, bearing three long plumose setae. Mesothorax with five ventral sclerites; mesal one wide and transverse, sinuate at posterior margin, bearing short bipectinate setae near midcoxae; antero-lateral ones oblong, bearing long bipectinate setae. Metathorax with five ventral sclerites; mesal one wide and transverse, bearing short setae. Granules on dorsal surface of thorax and abdomen (Fig. 7D, E) distributed linearly and somewhat irregularly, bearing short setae in posterior end, with long and curved setae in basal part. Y-shaped projections (Fig. 7A, D, F) present on caudal margins of thoracic and abdominal segments. Spiracles thumb-shaped, on mesothorax and abdominal segments I–VIII, situated near lateral margin. Legs 5-segmented, short and stout; apical segment stout, with short inner seta. Abdomen (Fig. 8C) with pleural sclerites on segments I–VII, gently tapering caudally. Abdominal segment IX (Fig. 8G) long, as long as abdominal segments VI–VIII combined, flat ventrally, serrate at apex (Fig. 8E), with line of granules in midline of dorsal surface, bearing four long lateral setae and one ventral seta, densely covered with short spines. Ventral operculum (Fig. 8G, H) oblong, situated in caudal 1/3 of abdominal segment IX.

#### Biological notes.

This species lives in small rivers at low elevation. Larvae and adults were collected from submerged roots of reeds using a net. They were collected with *Elmomorphus brevicornis* Sharp, 1888 and *Stenelmis nipponica* Nomura, 1958.

#### Distribution.

So far, this species is known only in the Iroha and Yakkan river systems, in the eastern part of Kyushu, Japan.

#### Etymology.

“Gyobu” is an NPO (nonprofit organization) in Kitakyushu, Fukuoka. This new species was discovered during a survey of water beetle fauna by Mr D. Inoue, president of “NPO Kitakyushu Gyobu”. The epithet is a noun in apposition.

### Key to Japanese genera of Macronychini (adult)

**Table:** 

1	Body oval, somewhat convex dorsally, shorter than 1.5 mm in TL	**2**
–	Body oblong, relatively flat dorsally, longer than 1.5 mm in TL	**4**
2	Antennae 8- or 7-segmented; humeri developed	**3**
–	Antennae 6-segmented; humeri reduced	*** Podonychus ***
3	Antennae 7-segmented; lateral margin of elytra serrate	*** Sinonychus ***
–	Antennae 8-segmented; lateral margin of elytra not serrate	*** Zaitzeviaria ***
4	Lateral declivity of pronotum with impressions	**5**
–	Lateral declivity of pronotum without impressions	*** Paramacronychus ***
5	Elytra with carinae on intervals V, VI, VII or V, VII, VIII	*** Zaitzevia ***
–	Elytra with carinae on only anterior part of intervals V and VI	*** Urumaelmis ***


### Key to Japanese genera of Macronychini (larva)^1^

**Table:** 

1	Dorsum with longitudinal stripes of granules; sides of thorax serrate; thorax and abdomen with Y-shaped projections attaching on caudal margins; apex of abdominal segment IX serrate	* Podonychus *
–	Dorsum without stripes of granules; sides of thorax not serrate; thorax and abdomen lacking Y-shaped projections; apex of abdominal segment IX with a small notch	2
2	Coloration of body entirely orange; abdominal segment IX distinctly convex ventrally	* Paramacronychus *
–	Coloration of body entirely pale brown, dark brown or black; abdominal segment IX flat ventrally	3
3	Cross-section of body triangular; coloration of body entirely pale brown	* Zaitzeviaria *
–	Cross-section of body semicircular; coloration of body entirely black or dark brown	* Zaitzevia *

## Discussion

*Podonychus* is currently known only from Indonesia and Kyushu, Japan, representing a disjunct distribution across the equator. We think this genus is probably distributed more widely in the Oriental Region but has not been found in other areas because of the small body size and unusual microhabitat. All specimens of the new species were collected from the submerged roots of reeds, and we think this microhabitat is important for the genus. In the future, additional species of this genus are expected to be discovered from east and southeast Asia, e.g., China, Taiwan, Vietnam, and the Philippines.

*Podonychus sagittarius* was collected from a small stream densely shaded by forest (Jäch and Kodada 1997), while the new species was collected from a small river running in open land (Fig. 1F). Judging from the long legs, both species live on submerged roots or rotten wood.

The endophallic structures of *Podonychus*, with a well sclerotized long apical tube, are unique not only in the tribe Macronychini, but also in the family Elmidae. The endophallic structures within the family are usually membranous as is probably the case in many Coleoptera (Dam 2014; Hayashi and Yoshitomi 2015). To obtain new relevant information about fine morphology, the endophallus must be fully everted for examination (Hayashi and Yoshitomi 2015).

The larval morphology of this genus is unique, particularly in having Y-shaped projections (Fig. 7A, E, D) on the thorax and abdomen. The characteristic of the larvae is basically similar to the other Macronychini genera (Hayashi and Sota 2010;Hayashi and Yoshitomi 2015), but the shape of the granules on the surface is similar to that of the larvae of *Pseudamophilus japonicus* (Hayashi and Sota 2010), which live on the surface of rotten wood or submerged roots of reeds in rivers. It is thought that the similarity of the shape of the granules on the surface of the larvae of *Podonychus* and *Pseudamophilus* is due to the larval microhabitat.

## Supplementary Material

XML Treatment for Podonychus

XML Treatment for Podonychus gyobu

## Footnotes

The larvae of Urumaelmis and Sinonychus are unknown.
